# In vitro metabolism, reaction phenotyping, enzyme kinetics, CYP inhibition and induction potential of ataluren

**DOI:** 10.1002/prp2.576

**Published:** 2020-03-20

**Authors:** Ronald Kong, Jiyuan Ma, Seongwoo Hwang, Young‐Choon Moon, Ellen M. Welch, Marla Weetall, Joseph M. Colacino, Neil Almstead, John Babiak, Elizabeth Goodwin

**Affiliations:** ^1^ PTC Therapeutics, Inc. South Plainfield NJ USA

**Keywords:** CYP Inhibition and CYP Induction, Enzyme Kinetics, In vitro Metabolism, Reaction Phenotyping, UGT

## Abstract

Ataluren promotes ribosomal readthrough of premature termination codons in mRNA which result from nonsense mutations. In vitro studies were performed to characterize the metabolism and enzyme kinetics of ataluren and its interaction potential with CYP enzymes. Incubation of [^14^C]‐ataluren with human liver microsomes indicated that the major metabolic pathway for ataluren is via direct glucuronidation and that the drug is not metabolized via cytochrome P450 (CYP). Glucuronidation was also observed in the incubation in human intestinal and kidney microsomes, but not in human pulmonary microsomes. UGT1A9 was found to be the major uridine diphosphate glucuronosyltransferase (UGT) responsible for ataluren glucuronidation in the liver and kidney microsomes. Enzyme kinetic analysis of the formation of ataluren acyl glucuronide, performed in human liver, kidney, and intestinal microsomes and recombinant human UGT1A9, found that increasing bovine serum albumin (BSA) levels enhanced the glucuronidation Michaelis‐Menten constant (K_m_) and ataluren protein binding but had a minimal effect on maximum velocity (V_max_) of glucuronidation. Due to the decreased unbound Michaelis‐Menten constant (K_m,u_), the ataluren unbound intrinsic clearance (CL_int,u_) increased for all experimental systems and BSA concentrations. Human kidney microsomes were about 3.7‐fold more active than human liver microsomes, in terms of CL_int,u_/mg protein, indicating that the kidney is also a key organ for the metabolism and disposition of ataluren in humans. Ataluren showed no or little potential to inhibit or induce most of the CYP enzymes.

**Abbreviations**
BSA: bovine serum albumin
CL_int__,u_: unbound intrinsic clearance
CL_int__,__u,UGT_: unbound intrinsic clearance by glucuronidation
C_max_: maximum plasma concentration
CYP: cytochrome P450
DMD: Duchenne muscular dystrophy
DMSO: dimethyl sulfoxide
EMA: European Medicines Agency
f_u__,inc_: fraction unbound from proteins in the incubation
HIM: human intestinal microsomes
HKM: human kidney microsomes
HLM: human liver microsomes
IC_50_: concentration of an inhibitor that causes a 50% decrease in enzyme activity
K_i_: inhibition constant that defines the affinity of a reversible inhibitor for an enzyme
K_i__,u_: unbound inhibition constant
K_m_: Michaelis‐Menten constant
K_m__,u_: unbound Michaelis‐Menten constant
LC/RAM/MS: liquid chromatography/radioactivity monitor/mass spectroscopy
LC‐MS/MS: liquid chromatography‐tandem mass spectrometry
MTT: methylthiazolyldiphenyl‐tetrazolium bromide
nmDMD: nonsense mutation Duchenne muscular dystrophy
RAM: radioactivity monitor
UDPGA: uridine diphosphate glucuronic acid
UGT: uridine diphosphate glucuronosyltransferase
V_max_: maximum velocity

## 1 INTRODUCTION

**Figure 1:**
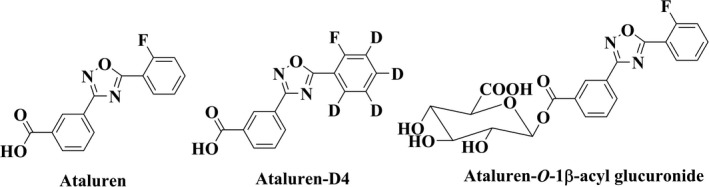
Structures of ataluren, ataluren‐D4 and ataluren‐*O*‐1β‐acyl glucuronide

Ataluren (PTC124, Translarna™, Figure 1) is a small molecule drug that is being developed for the treatment of genetic disorders resulting from nonsense mutations. Nonsense mutations, which encode premature stop codons, result in the premature termination of protein translation in the coding region of an mRNA.^1^ Ataluren promotes ribosomal read‐through of a premature termination codon in the dystrophin mRNA, a cause of Duchenne muscular dystrophy, resulting in production of full‐length protein.^1^, ^2^, ^3^, ^4^, ^5^

Duchenne muscular dystrophy (DMD) is an X‐linked genetic muscle disorder that results from the presence of mutations in the gene that encodes the dystrophin protein. Dystrophin provides stability to the muscle and acts as a shock absorber, bearing the mechanical stresses that occur during contraction, stabilizing the cell membranes, and protecting the muscle from injury.^6^, ^7^, ^8^, ^9^ Approximately, 10% to 15% of boys with DMD have the disease due to nonsense mutations.^10^, ^11^, ^12^

Ataluren has shown to produce full length, functional dystrophin (^2^ ^,^ ^4^ ^,^ ^13^) in the nonsense mutation mdx mouse and sapje zebrafish models of DMD. Ataluren activity has also been demonstrated in multiple cell‐based and animal disease models of other nonsense mutation genetic disorders, corroborating its ability to promote readthrough of premature stop codons and its potential for treating genetic disease caused by nonsense mutations.^14^, ^15^, ^16^, ^17^, ^18^, ^19^ Comprehensive nonclinical studies have been conducted in safety pharmacology and secondary pharmacodynamics, pharmacokinetics and metabolism, and toxicology programs. Following a single oral dose of [^14^C]‐ataluren in mice, rats, dogs, and humans, ataluren was well absorbed and cleared primarily by metabolism. Biliary secretion was the major route for elimination of drug‐related radioactivity in bile‐duct cannulated rats. Ataluren acyl glucuronide was the only detectable metabolite in human plasma, and the major metabolic and clearance pathways in humans are similar to animal species.^20^ The acceptable pharmacokinetic, toxicokinetic, and safety profiles of ataluren support the use of ataluren for chronic administration to patients with genetic disorders resulting from nonsense mutations.

Ataluren clinical development program consists of Phase 1 studies characterizing the absorption, metabolism, and excretion profile of ataluren and evaluating the pharmacokinetics and safety profiles in healthy subjects,^20^, ^21^, ^22^ Phase 2 studies in patients with nonsense mutation genetic disorders,^5^ and long‐term Phase 2b/3 studies in patients with nonsense mutation Duchenne muscular dystrophy (nmDMD) and nonsense mutation cystic fibrosis (nmCF).^23^, ^24^, ^25^ Ataluren has shown good tolerability in healthy subjects and patients, and has demonstrated clinical benefit in two multicenter, randomized, clinical trials to slow disease progression in patients with nmDMD.^24^ ^,^ ^25^ On this basis, ataluren received conditional marketing authorization for the treatment of nmDMD in ambulatory patients aged 5 years and older in the European Union, marketing authorization for the treatment of nmDMD in Israel, conditional approval for treatment of nmDMD in South Korea, marketing approval for the treatment of ambulatory children five years and older with nmDMD in Brazil, and most recently in Kazakhstan. Phase 3 trials in nmDMD are ongoing, as are Phase 2 studies in nonsense mutation Dravet syndrome/cyclin‐dependent kinase‐like 5 (CDKL5) and nonsense mutation Aniridia. The clinical development of ataluren in nmCF was discontinued.

Extensive in vitro and in vivo studies have been conducted to characterize absorption, distribution, metabolism, excretion, pharmacokinetic/toxicokinetic properties, and drug interaction potentials of ataluren. Most recent publications include ataluren pharmacokinetics in Japanese healthy subjects,^22^ and the absorption, metabolism, and excretion following a single oral dose of [^14^C]‐ataluren in mice, rats, dogs, and humans.^20^ The additional manuscripts in preparation include in vitro and in vivo stability assessment of ataluren acyl glucuronide, evaluation of drug metabolizing enzyme‐ and transporter‐mediated drug‐drug interaction potentials in healthy subjects, pharmacokinetics evaluation of ataluren in special population, and safety, pharmacokinetic, and pharmacodynamic assessments in nmDMD patients aged ≥ 2 to < 5 years, etc. The purpose of the current manuscript is to describe the in vitro metabolism and enzyme kinetics of ataluren and its interaction with CYP enzymes. The results presented here have provided the understanding of ataluren disposition and metabolism in vitro and informed the need and design of additional clinical studies to investigate the potential drug‐drug interactions in human.

## 2 MATERIALS AND METHODS

### 2.1 Materials

Ataluren was manufactured by Siegfried (Zofingen, Switzerland) and [^14^C]‐ataluren (99% radio‐ and chemical purity) was prepared by ABC Laboratories (Columbia, MO, USA). Ataluren‐D_4_ and ataluren‐*O‐*1β‐acyl glucuronide were synthesized by PTC Therapeutics (South Plainfield, NJ, USA). Plasma from male C57/BL10 mice, Sprague Dawley rats, beagle dogs, cynomolgus monkeys, and humans was purchased from Biochemed Pharmacologicals. Pooled human liver microsomes (HLM) were obtained from XenoTech LLC, Celsis‐In Vitro Technologies, or Tissue Transformation Technologies. Individual HLM (reaction phenotyping kit from 16 individual donors), pooled human kidney (prepared from both cortex and medulla), pooled human pulmonary and pooled human intestine microsomes were provided by XenoTech LLC. Fresh human hepatocytes and media for CYP induction were obtained from Celsis‐In Vitro Technologies (Baltimore, MD, USA). Recombinant human UGT enzymes expressed in baculovirus‐infected insect cells were obtained from BD Biosciences. CYP substrates, metabolites, and inhibitors were obtained from BD Biosciences, Fisher Scientific, or XenoTech LLC. All other chemical reagents were purchased from Sigma‐Aldrich Chemical Co.

## 3 METHODS

### 3.1 In vitro metabolism and plasma protein binding

#### 3.1.1 In vitro* metabolism*

[^14^C]‐Ataluren at 1, 10, and 100 μmol/L was incubated (n = 3) with pooled HLM (1 mg protein/mL) at 37 ± 1°C in 0.2 mL incubation mixtures (final volume) containing potassium phosphate buffer (50 mmol/L, pH 7.4), MgCl_2_ (3 mmol/L), and EDTA (1 mmol/L) with and without cofactors, NADPH‐generating system (1 mmol/L NADP, 5 mmol/L glucose‐6‐phosphate, and 1 unit/mL glucose‐6‐phosphate dehydrogenase), and uridine diphosphate glucuronic acid (UDPGA, 8 mmol/L). [^14^C]‐Ataluren was added in DMSO (final percent of 0.1% v/v). Reactions were started by the addition of the cofactors and terminated by the addition of stop reagent (0.2 mL of 2% v/v formic acid/acetonitrile) at 0, 60, and 120 minutes. Precipitated protein was removed by centrifugation (920 *g* for 10 minutes at 10°C). The supernatant fractions were analyzed by LC/RAM/MS to determine percent loss of substrate in incubations. Detailed LC/MS conditions are summarized in LC‐MS/MS Analysis section. After chromatographic separation, the detected radioactive peaks were integrated, and their identity was confirmed using mass spectrometry analysis. [^14^C]‐ataluren remaining at specific timepoint after incubation was compared with the amount of [^14^C]‐ataluren at 0 minutes incubation sample in the same matrix (eg, with or without cofactors etc) to calculate the percentage of ataluren remaining. The percentage of [^14^C]‐ataluren acyl glucuronide formation at specific timepoint after incubation was calculated using the amount of metabolite formed over the total radioactivity in each sample.

#### 3.1.2 Plasma protein binding

[^14^C]‐Ataluren, at 50, 500, 5000, and 50 000 ng/mL or 0.175, 1.75, 17.5, and 175 µM, in male mouse, rat, dog, monkey, and human plasma, was incubated (n = 3) at 37°C for approximately 15 minutes, transferred into Centrifree^®^ Micropartition Units (30 000 Da molecular weight cutoff, 1 mL capacity; Amicon Inc), and centrifuged at 37°C and 2000 *g* for approximately 10 minutes so that the amount of the sample filtered was approximately ≤ 20% of the total volume. After centrifugation, the entire ultrafiltrate was weighed and analyzed for radioactivity using liquid scintillation counting. The percent bound = (1‐C_u_/C_m_)×100, where C_u_ is the concentration of radioactivity in the ultrafiltrate and C_m_ is the concentration of radioactivity in the plasma before centrifugation.

### 3.2 Reaction phenotyping and kinetics for the formation of ataluren acyl glucuronide

#### 3.2.1 Recombinant human UGT enzymes

Ataluren at 10 μmol/L, was incubated (n = 2) with recombinant human uridine diphosphate glucuronosyltransferase (rUGT) enzymes: rUGTs 1A1, 1A3, 1A4, 1A6, 1A7, 1A8, 1A9, 1A10, 2B4, 2B7, 2B15, and 2B17 at 0.25 mg protein/mL. Incubations were conducted in duplicate at 37°C. The incubation mixture (0.2 mL, final volume with 1% methanol) consisted of Tris‐HCl buffer (100 mmol/L, pH 7.7 at 37°C), MgCl_2_ (10 mmol/L), EDTA (1 mmol/L), saccharic acid 1,4‐lactone (0.1 mmol/L), and UDPGA (8 mmol/L). Alamethicin was prepared in 20 mmol/L EDTA and the activation of the recombinant UGT enzymes was performed by adding an equal volume of the protein and alamethicin solution to achieve 25 μg alamethicin/mg protein. The protein/alamethicin mixture was preincubated for 15 minutes on ice, prior to the addition of ataluren to the mixture. Ataluren was added in methanol (1% v/v). Reactions were initiated by the addition of UDPGA and were terminated at 0 and 60 minutes by the addition of 2% v/v formic acid in acetonitrile containing ataluren‐D4 as an internal standard. Samples were subjected to centrifugation (920 *g* for 10 minutes at 10°C) and the supernatant fractions were analyzed by LC‐MS/MS to quantify the formation of ataluren‐*O‐*1β‐acyl glucuronide and loss of ataluren. Detailed LC‐MS/MS conditions are summarized in LC‐MS/MS Analysis section. The amount of ataluren remaining after 60 minutes incubation was compared with the amount of ataluren at 0 minutes incubation sample in the same matrix to calculate the percentage of ataluren remaining. The ataluren acyl glucuronide formation rate after 60 minutes incubation was calculated using the amount of metabolite formed (pmol) divided by the protein amount in the incubation sample (0.05 mg) and the incubation time (60 minutes).

#### 3.2.2 Correlation analysis

Ataluren at a concentration of 10 μmol/L, was incubated (n = 2) with 16 individual HLM at 0.5 mg protein concentration/mL for 0 and 60 minutes at 37°C. Enzyme activities for UGTs 1A1, 1A4, 1A6, 1A9, and 2B7 were precharacterized under optimized conditions by the vendor. Incubation mixtures and procedures were essentially the same as the above. The supernatant fractions were analyzed by LC‐MS/MS to quantify the formation of ataluren‐*O‐*1β‐acyl glucuronide and the loss of ataluren, and the enzyme activities were correlated with precharacterized UGT enzyme activities. Detailed LC‐MS/MS conditions are summarized in LC‐MS/MS Analysis section. The amount of ataluren remaining after 60 minutes incubation was compared with the amount of ataluren at 0 minutes incubation sample in the same matrix to calculate the percentage of ataluren remaining. The ataluren acyl glucuronide formation rate after 60 minutes incubation was calculated using the amount of metabolite formed (pmol) divided by the protein amount in the incubation sample (0.2 mg) and the incubation time (60 minutes). Differences in the rate of the formation of ataluren acyl glucuronide were compared with the sample‐to‐sample variation on the activities for UGTs 1A1, 1A4, 1A6, 1A9, and 2B7 that were precharacterized under optimized conditions by the vendor. Correlation analysis was performed by linear regression with a laboratory information management system (LIMS, Galileo, version 3.3). Graphs were prepared using LIMS or the computer software program, Microsoft Excel 2003 (Microsoft Corp.).

#### 3.2.3 Incubations of ataluren with microsomes from extrahepatic tissues

Ataluren at 10 μmol/L, was incubated (n = 1) with pooled human intestinal, lung, and kidney microsomes (1 mg protein/mL, preactivated with alamethicin) to evaluate if ataluren‐*O‐*1β‐acyl glucuronide will be formed in these extrahepatic tissues. Incubation mixtures and procedures were essentially the same as the above. The supernatant fractions were analyzed by LC‐MS/MS to quantify the formation of ataluren‐*O*‐1β‐acyl glucuronide and the loss of ataluren. Detailed LC‐MS/MS conditions are summarized in LC‐MS/MS Analysis section. The amount of ataluren remaining after 60 minutes incubation was compared with the amount of ataluren at 0 minutes incubation sample in the same matrix to calculate the percentage of ataluren remaining. The ataluren acyl glucuronide formation rate after 60 minutes incubation was calculated using the amount of metabolite formed (pmol) divided by the protein amount in the incubation sample (0.4 mg) and the incubation time (60 minutes).

#### 3.2.4 Enzyme kinetics for the formation of ataluren acyl glucuronide

Ataluren at 0.5, 2.5, 12.5, 25, 50, 100, 200, and 400 µmol/L, was incubated (n = 3) at 37°C with recombinant human UGT1A9 and with pooled microsomes from human liver, kidney, and intestine (0.025 mg protein/mL, preactivated with alamethicin at 20 µg/mL) in Tris‐HCl buffer (0.1 mol/L, pH 7.5), in the presence of 1 mmol/L EDTA, 5 mmol/L MgCl_2_, with or without 0.5%, 1%, or 2% BSA. The incubation (100 µL final volume with 0.5% DMSO) was started by adding an equal volume of UDPGA (5 mmol/L) in Tris‐HCl buffer (0.1 mol/L, pH 7.5). After 30 minutes incubation, the incubation mixtures were combined with 0.3 mL of an internal standard solution in acetonitrile with 1% formic acid, followed by membrane plate filtration. The filtrates were used for LC‐MS/MS analysis to quantify the formation of ataluren‐*O‐*1β‐acyl glucuronide. Detailed LC‐MS/MS conditions are summarized in LC‐MS/MS Analysis section. Ataluren acyl glucuronide formation rate after 30 minutes incubation was calculated using the amount of metabolite formed (pmol) divided by the protein amount in the incubation sample (0.0025 mg) and the incubation time (30 minutes). Ataluren glucuronidation kinetic parameters were determined with and without adjusting BSA binding by nonlinear regression (GraphPad Prism, 5version 5.04, GraphPad Software, Inc.) using Michaelis‐Menten equation which was the best fit compared with other models. The intrinsic clearance was calculated as CL_int_ = V_max_/K_m_. In order to compare unbound intrinsic clearance by glucuronidation (CL_int,u,UGT_) in different tissues, CL_int,u,UGT_ was expressed per gram of tissue by correcting the values for the microsomal protein yield, resulting in scaled CL_int,u,UGT_. Microsomal protein yields of 40.0, 12.8, and 20.6 mg protein/g tissue were used for hepatic, renal, and intestinal data, respectively. ^26^

#### 3.2.5 Correction for BSA binding

The high‐throughput membrane ultrafiltration method ^27^ was used to determine the fraction unbound in the incubation (n = 3). Incubation mixtures and procedures were essentially the same as the above except that UDPGA (5 mmol/L) was replaced with Tris‐HCl buffer (0.1 mol/L, pH 7.5). After 15 minutes incubation, aliquot was transferred to a Millipore Multiscreen Ultracel‐10 filter plate (10 kDa molecular mass cut off; EMD Millipore). The plate was centrifuged at 37°C and 3710 *g*. After 30 minutes centrifugation, aliquots of ultrafiltrates and incubation mixtures prior to ultrafiltration were quenched with an internal standard solution in acetonitrile, followed by membrane plate filtration. The filtrates were used for LC‐MS/MS analysis to quantify the concentration of ataluren. The same LC‐MS/MS conditions described above are summarized in LC‐MS/MS Analysis section. The free fraction = C_u_/C_m_, where C_u_ is the concentration of ataluren in the ultrafiltrate and C_m_ is the concentration of ataluren in the incubation system before centrifugation. Unbound K_m_ (K_m_, u) was recalculated by applying Michaelis‐Menten equation using free ataluren concentrations after correcting BSA binding at each ataluren and BSA concentration level.

### 3.3 CYP inhibition and induction

#### 3.3.1 CYP inhibition

The metabolic reactions that were monitored and probe substrate concentrations used are shown in Table S1. The final concentration of each probe substrate was near the experimentally determined K_m_ value for the indicated enzyme. Ataluren or prototypical CYP inhibitors (positive controls) was incubated (n = 3) with probe substrates and HLM in 100 mmol/L phosphate buffer (pH 7.4) in a total volume of approximately 0.25 mL, and the reactions were initiated by the addition of 1 mmol/L NADPH at 37°C. The reactions were carried out using previously established conditions to ensure linearity with respect to protein concentration and incubation time (Table S1). Incubations were stopped with the addition of ice‐cold acetonitrile. Metabolite formation in incubations with test compound and control inhibitors was assessed with LC‐MS/MS methods for each of the reaction products as described in Table S2. For inhibition constant (*K*_i_) determination, ataluren, at 0, 2.5, 12.5, 25, 50, 100, 200, and 400 µmol/L, and known CYP inhibitors at various concentrations that bracket reported *K*_i_ values for each CYP enzyme, were separately incubated (n = 3) with human liver microsomes (0.1 mg/mL protein concentration) in potassium phosphate buffer (0.1 mol/L, pH 7.4), in the presence of 1 mmol/L EDTA, 3 mmol/L MgCl_2_, NADPH (1.3 mmol/L NADP, 3.3 mmol/L glucose‐6‐phosphate, and 1 unit/mL glucose‐6‐phosphate dehydrogenase), and the respective CYP marker substrate at various concentrations (approximately, <1/3 *K*_m_ to > 3 *K*_m_). After 7 minutes incubation at 37°C, the reaction was stopped by the addition of ice‐cold acetonitrile, followed by membrane plate filtration. The filtrates were used for LC‐MS/MS analysis. Enzyme activity remaining at each nonzero ataluren concentration after incubation was normalized against control sample (zero ataluren concentration, enzyme activity = 100%), and the % remaining enzyme activity values were used for IC_50_ calculation. IC_50_ values were determined by nonlinear regression with GraphPad Prism 5 using the following four‐parameter sigmoidal‐logistic IC_50_ equation:

$$\text{Fit}=\text{background}+\left(\text{range}-\text{background}\right)/\left[1\;+\;{\left(I/{\text{IC}}_{50}\right)}^{\text{slope}}\right]$$

Where, I is the inhibitor concentration, and background was set to 0 and range to 100, as percent of control values are utilized. *K*_i_ was determined by nonlinear regression with GraphPad Prism 5 using best fit model.

#### 3.3.2 CYP induction

Ataluren, at 4, 40, and 400 µmol/L, and prototypic CYP inducers at their validated concentrations, omeprazole at 50 µmol/L, phenobarbital at 1 mmol/L, and rifampin at 25 µmol/L were incubated with freshly isolated human hepatocytes. Human hepatocytes isolated from three Caucasian males were transferred to 48‐well collagen‐coated plates at 0.14 × 10^6^ cells/well in planting media for attachment. After attachment, planting media was replaced with sandwich media and the cells were incubated until clusters were established. Subsequently, the sandwich medium was removed, and the hepatocytes were treated with incubation solution containing ataluren (4, 40, and 400 µmol/L), solvent control (1% DMSO), omeprazole (50 µmol/L), phenobarbital (1 mmol/L), or rifampin (25 µmol/L) for 24 hours (n = 3 for ataluren at each concentration; n = 4 for solvent and positive controls). The incubation solutions were aspirated and replaced with freshly prepared incubation solutions containing ataluren for an additional 24 hours. After total of 48 hours treatment, the incubation solutions were replaced with Krebs‐Henseleit (K‐H) buffer and incubated for 10 minutes to remove residual ataluren. The incubation solutions were then aspirated and 150 µL of K‐H buffer containing CYP substrates were added. The hepatocytes were incubated for 30 minutes for CYP2C8 (10 µmol/L amodiaquine), 1 hour for CYP3A4 (125 µmol/L testosterone), and 4 hours for CYPs 1A2 (100 µmol/L phenacetin), 2A6 (100 µmol/L coumarin), 2B6 (150 µmol/L bupropion), 2C9 (50 µmol/L tolbutamide), 2C19 (100 µmol/L *S*‐mephenytoin), and 2E1 (300 µmol/L chlorzoxazone) activity assays. The reactions were stopped by the addition of 0.15 mL of ice‐cold methanol except that coumarin‐treated cells were incubated with β‐glucuronidase and aryl sulfatase for additional 1 hour to free 7‐hydroxycoumarin before the addition of 0.2 mL of ice‐cold methanol. Metabolite formation in incubations with test compounds and control inhibitors was assessed with validated LC‐MS/MS methods for each of the reaction products as described in Table S2. Enzymatic activity for each CYP was calculated using the absolute amount of metabolite formed (pmol) divided by hepatocytes in the incubation (million cells) and the incubation time (minute). The relative fold induction in enzymatic activity was calculated by comparing the rate of metabolite formation for treatment groups to that of the solvent control group (reported as vehicle control%) or positive control group (reported as positive control%).

### 3.4 LC‐MS/MS analysis

#### 3.4.1 Metabolism of Ataluren in HLM

[^14^C]‐Ataluren and metabolites were monitored by LC/RAM/MS system, which consisted of two Shimadzu LC‐10ATvp pumps (Shimadzu Scientific Instruments, Columbia, MD, USA), a Shimadzu SIL‐5000 autosampler, an IN/US Systems β‐RAM radiometric detector (LabLogic), and a Sciex API 4000 QTRAP triple quadrupole mass spectrometer (Sciex) equipped with a Turbo Ionspray ionization source operated in negative ionization mode. The Ionspray voltage was set at 4.5 kV with the source temperature at 450ºC. MS/MS was carried out with nitrogen as the collision gas. The column used was a Phenomenex Luna C8, 3 μm, 150 × 3.0 mm column (Phenomenex, Torrance, CA, USA). Mobile phase A consisted of 0.2% formic acid in water and mobile phase B consisted of 0.2% formic acid in acetonitrile. The flow rate was 0.50 mL/min with gradient program as follows: hold isocratic at 5% B for 2 minutes; linear gradient from 5% B to 60% B in 20 minutes; linear gradient from 60% B to 95% B in 5 minutes and hold isocratic at 95% B for 2 minutes; linear gradient from 95% B to 5% B in 0.5 minutes and hold isocratic at 0% B for 3.5 minutes.

#### 3.4.2 Reaction phenotyping for the formation of ataluren acyl glucuronide

Quantification of ataluren loss and ataluren‐*O‐*1β‐acyl glucuronide formation was carried out with an LC‐MS/MS system, which consisted of two Shimadzu LC‐10ADvp pumps, a SPD10‐AVP UV/Vis detector, a Shimadzu SILHTA autosampler, a Shimadzu DGU‐14A degasser, and a Sciex API 4000 QTRAP mass spectrometer equipped with a Turbo Ionspray ionization source that operated in positive ionization mode. The Ionspray voltage was set at 4.5 kV with the source temperature at 550°C. The column used was a Phenomenex Luna C18 (100 × 4.6 mm, 3 μm) column. Mobile phase A was 0.2% formic acid in water, and mobile phase B was 0.2% formic acid in acetonitrile. The flow rate was 0.60 mL/min with gradient program as follows: hold isocratic at 5% B for 3 minutes; linear gradient from 5% B to 38% B in 6 minutes and hold isocratic at 38% for 1 minute; linear gradient from 38% B to 95% B in 3 minutes and hold isocratic at 95% B for 1 minute; linear gradient from 95% B to 5% B in 0.1 minutes and hold isocratic at 0% B for 5.9 minutes. Ion transitions monitored were at *m*/*z* 285 → *m*/*z* 123 for ataluren, *m*/*z* 461 → *m*/*z* 285 for ataluren acyl glucuronide, and *m*/*z* 289 → *m*/*z* 127 for ataluren‐D_4_ (internal standard), respectively.

#### 3.4.3 Kinetics for the formation of ataluren acyl glucuronide

Quantification of ataluren‐*O‐*1β‐acyl glucuronide formation was conducted on an Accela pump and a PAL autosampler equipped with a TSQ Quantum Ultra mass spectrometer (Thermo Fisher Scientific). A Waters Acquity BEH column (C18, 1.7 µm, 50 × 2.1 mm; Waters Cooperation) was used and maintained at 50°C. The mobile phases were 0.1% formic acid in water (A) and acetonitrile (B). The flow rate was 0.6 mL/min and gradient program was as follows: linear gradient from 0% B to 95% B in 0.5 minutes and hold isocratic at 95% B for 1 minute; linear gradient from 95% B to 0% B in 0.1 minutes and hold isocratic at 0% B for 0.4 minutes. TSQ Quantum Ultra mass spectrometer was equipped with a heated electrospray ionization source operated in negative‐ion mode with a vaporizer temperature of 350°C, capillary temperature of 380°C, and spray voltage of 3 kV. The sheath, auxiliary, and sweep gas pressure were 60, 50, and 3 units, respectively. Ion transitions monitored were at *m*/*z* 283 → *m*/*z* 163 for ataluren, *m*/*z* 459 → *m*/*z* 283 for ataluren acyl glucuronide, 287 → *m*/*z* 167 for ataluren‐D_4_ (internal standard), respectively, with normalized collision energy at 20%.

#### 3.4.4 Data analysis

The study design was not powered for statistical analysis. All assays were run in singlet, duplicate or triplicate and were used only to test precision of n = 1 and not analyzed as independent experiments. The number of replicates in each experiment is shown in experimental methods section as well as in relevant tables and figures. Calibration standards were employed to calculate concentration based on analyte/internal standard peak‐area ratios using Analyst, Xcalibur MS System software, or equivalent, to quantify metabolite formation and the amount of test article remaining in the incubation.

## 4 RESULTS

### 4.1 Metabolism of ataluren in human liver microsomes

[^14^C]‐Ataluren at concentrations of 1, 10, and 100 µmol/L, was incubated with pooled HLM with different cofactors for up to 120 minutes. However, no ataluren loss was observed and no metabolites were detected after incubation of [^14^C]‐ataluren with NADPH fortified HLM, whereas 32%, 31%, and 18% ataluren loss was observed after 120 minutes at 1, 10, and 100 µmol/L (n = 3), respectively, and [^14^C]‐ataluren‐*O‐*1β‐acyl glucuronide was the only metabolite found after incubation of [^14^C]‐ataluren with UDPGA fortified HLM.

### 4.2 Plasma protein binding

The percentage protein binding of [^14^C]‐ataluren (mean ± standard deviation, n = 3) was 98.4 ± 0.5, 98.7 ± 0.2, 97.5 ± 0.2, 99.7 ± 0.1, and 99.6 ± 0.1 in mouse, rat, dog, monkey, and human plasma, respectively. No marked concentration‐dependent binding over the target concentration range of 50 to 50 000 ng/mL (0.175 to 175 µmol/L) in plasma from all species investigated was observed.

### 4.3 Reaction phenotyping for ataluren glucuronidation

**Figure 2:**
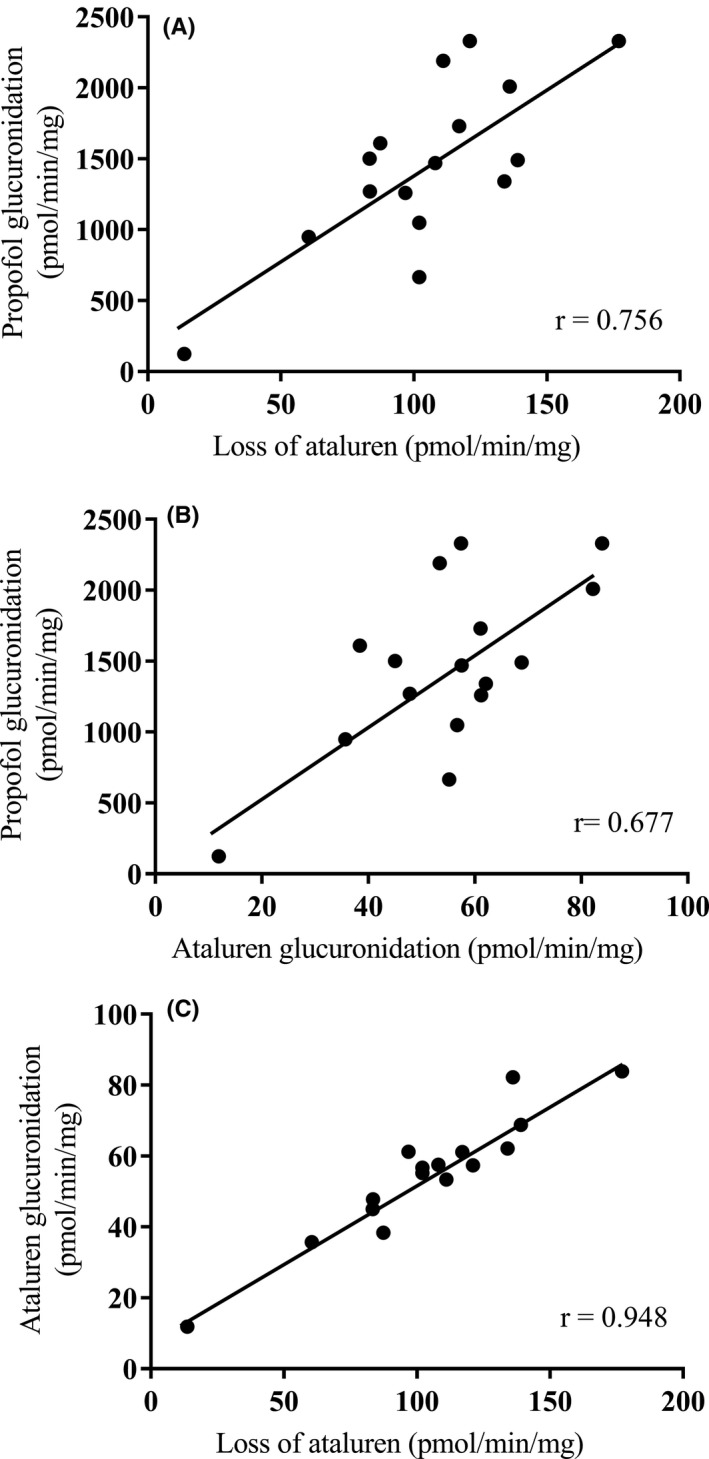
Correlation between the rate of disappearance of ataluren, the rate of the acyl glucuronide formation, and marker UGT enzyme activity in a bank (n = 16) of individual human liver microsomes (n = 2 for each data point). (A) loss of ataluren vs propofol glucuronidation, (B) ataluren glucuronidation vs propofol glucuronidation, and (C) loss of ataluren vs ataluren glucuronidation

The greatest loss of ataluren and the greatest formation of ataluren‐*O‐*1β‐acyl glucuronide was seen with recombinant human UGT1A9 (32.7% ataluren loss and 187 pmol/mg/min ataluren glucuronide formation after 60 minutes incubation, n = 2) followed by UGT1A7 (6.2% ataluren loss and 41.8 pmol/mg/min ataluren glucuronide formation after 60 minutes incubation, n = 2). The loss of ataluren and the formation of ataluren‐*O*‐1β‐acyl glucuronide correlated significantly with UGT1A9 activity, as measured by glucuronidation of propofol, with correlation coefficients (r) of 0.756 and 0.677, respectively. The loss of ataluren and ataluren acyl glucuronide formation significantly correlated with one another (r = 0.948) confirming that the same enzyme (UGT1A9) was responsible for both the loss of ataluren and ataluren acyl glucuronide formation (Table 1 and Figure 2). The loss of ataluren and the formation of ataluren‐*O‐*1β‐acyl glucuronide did not correlate significantly with any of the other enzyme activities evaluated (ie, UGTs 1A1, 1A3, 1A4, 1A6, 1A7, 1A8, 1A10, 2B4, 2B7, 2B15, and 2B17). Since UGT1A7 is not expressed in human liver, glucuronide formation and substrate loss could not be correlated with UGT1A7 activity (Table 1).

**Table 1:** Analysis of the correlation between the rate of disappearance of ataluren, the rate of the glucuronide formation, and marker UGT enzyme activity in a bank (n = 16) of individual human liver microsomes

Human sample	Rate (pmol/mg/min, n = 2)								
	Loss of ataluren	Ataluren glucuronide formation	UGT1A1 (3‐gluc. of 17β‐estradiol)	UGT1A4 (gluc. of trifluoperazine)	UGT1A6 (gluc. of 1‐naphthol)	UGT1A9 (gluc. of propofol)	UGT2B7 (3‐gluc. of morphine)	UGT2B7 (6‐gluc. of morphine)	Unknown UGT (17β‐gluc. of 17β‐estradiol)
H0196	136	82.2	1180	1560	11 800	2010	7330	1170	97.8
H0245	96.8	61.2	395	1180	6160	1260	6580	1070	180
H0258	83.5	47.8	226	1260	6520	1270	6350	1160	83.3
H0259	139	68.8	719	1040	6490	1490	3060	541	24.3
H0273	111	53.4	306	745	9970	2190	4530	719	169
H0276	121	57.4	267	1040	7040	2330	5400	944	78.5
H0277	102	55.2	671	1380	7170	665	7450	1210	173
H0287	87.4	38.4	703	654	7230	1610	4170	717	67.5
H0288	108	57.5	487	1240	7090	1470	8090	1390	213
H0290	117	61.1	1360	1740	6840	1730	5630	966	89.4
H0292	177	83.9	995	1750	5590	2330	4280	815	57.9
H0295	134	62.1	718	733	5860	1340	5090	967	70.0
H0297	60.5	35.7	852	1010	4050	950	5210	945	63.9
H0298	83.4	45.0	506	947	6580	1500	5040	877	131
H0300	102	56.7	661	1950	8990	1050	9670	1750	122
H0305	13.7	11.9	447	1130	6360	123	4300	757	43.3

Activity	Enzyme	Loss of ataluren		Ataluren glucuronide formation	
		Correlation coefficient	*P* value	Correlation coefficient	*P* value
3‐Glucuronidation of 17β‐estradiol	UGT1A1	0.383	.143	0.430	.096
Glucuronidation of trifluoperazine	UGT1A4	0.279	.295	0.428	.098
Glucuronidation of 1‐naphthol	UGT1A6	0.220	.412	0.328	.215
Glucuronidation of propofol	UGT1A9	0.756	.001	0.677	.004
3‐Glucuronidation of morphine	UGT2B7	−0.010	.970	0.176	.514
6‐Glucuronidation of morphine	UGT2B7	−0.004	.989	0.154	.570
17β‐Glucuronidation of 17β‐estradiol	Unknown UGT	−0.004	.988	0.109	.687

Abbreviations: Gluc, glucuronide; UGT, uridine diphosphate glucuronosyltransferase.

Glucuronidation of ataluren was observed in kidney and intestine as well as in liver microsomes, but not in lung microsomes. After 60 minutes incubation, ataluren loss was 31.4%, 94.3%, and 34.4% (n = 1), and ataluren glucuronide formation rate was 62.0, 48.4, and 29.0 pmol/mg/min (n = 1) in liver, kidney, and intestinal microsomes, respectively.

### 4.4 Ataluren glucuronidation kinetics

**Figure 3:**
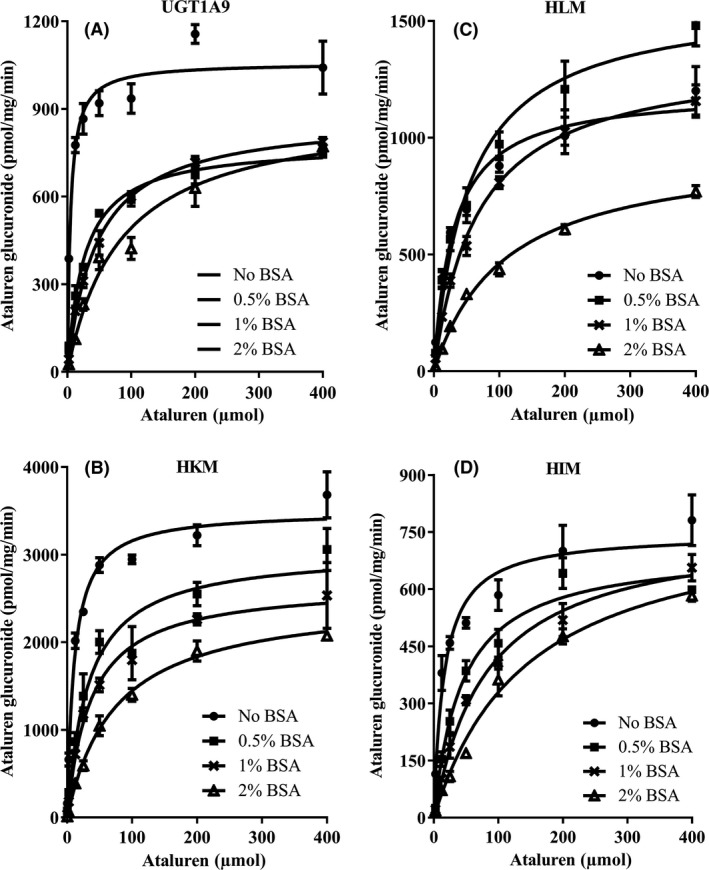
Enzyme kinetic (Michaelis‐Menten) plots for ataluren glucuronidation with or without the presence of BSA in (A) recombinant human UGT1A9, (B) human kidney microsomes, (C) human liver microsomes, and (D) human intestinal microsomes (n = 3 for each data point)

**Figure 4:**
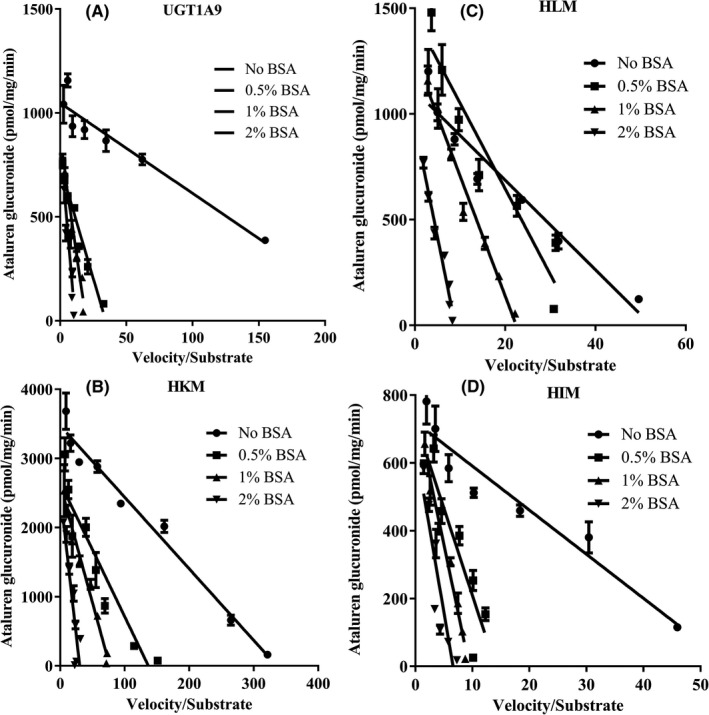
Eadie‐Hofstee plots for ataluren glucuronidation kinetics with or without the presence of BSA in (A) recombinant human UGT1A9, (B) human kidney microsomes, (C) human liver microsomes, and (D) human intestinal microsomes (n = 3 for each data point)

The results are shown in Table 2, Figure 3 and Figure 4. In general, the addition of BSA in the incubation system increased total K_m_ values and decreased K_m,u_ values in a BSA concentration‐dependent manner; effect of BSA on the enzyme velocity is system dependent: slightly decreased for HKM and HLM, whereas comparable for recombinant human UGT1A9 and HIM. However, BSA increased the CL_int,u_ with maximal effect at 1% BSA in all incubation systems. Without BSA, nonspecific binding of ataluren to proteins present in the incubation systems is minimal since very low protein concentration, 0.025 mg/mL, was used in all incubation systems. However, with BSA in the incubation system, binding of ataluren to BSA was significant and was dependent on both concentrations of BSA and ataluren (Table 2).

**Table 2:** Ataluren glucuronidation kinetic parameters in recombinant human UGT1A9 and in microsomes from human liver, kidney, and intestine in the presence of 0, 0.5, 1, and 2% BSA (n = 3)

System	UGT1A9				HLM				HKM				HIM			
BSA (%)	0	0.5	1	2	0	0.5	1	2	0	0.5	1	2	0	0.5	1	2
*V*_max_ ^a^ (pmol/min/mg)	1058 ± 31.8	752 ± 20.3	842 ± 23.0	847 ± 56.2	1204 ± 49.0	1465 ± 68.2	1264 ± 53.5	886 ± 34.7	3948 ± 90.3	2941 ± 168	2563 ± 137	2342 ± 119	747 ± 32.7	660 ± 28.3	711 ± 31.2	767 ± 54.6
*K*_m_ ^a^ (µmol/L)	4.76 ± 0.934	26.4 ± 2.80	45.6 ± 3.83	83.4 ± 15.3	29.9 ± 4.49	54.3 ± 9.42	67.9 ± 9.19	108 ± 10.7	10.8 ± 1.40	33.0 ± 7.36	36.5 ± 6.31	74.1 ± 8.38	15.8 ± 3.35	43.8 ± 6.88	82.4 ± 11.3	145 ± 22.0
f_u,inc_ ^b^	1.00 ± 0.50	0.116 ± 0.019	0.064 ± 0.007	0.035 ± 0.003	1.00 ± 0.120	0.093 ± 0.006	0.050 ± 0.007	0.030 ± 0.005	1.00 ± 0.121	0.095 ± 0.003	0.050 ± 0.002	0.030 ± 0.003	1.00 ± 0.137	0.100 ± 0.0021	0.056 ± 0.002	0.036 ± 0.002
*K*_m, u_ ^a^ (µmol/L)	4.76 ± 0.934	2.71 ± 0.309	2.52 ± 0.243	2.46 ± 0.498	29.9 ± 4.49	4.50 ± 0.748	3.00 ± 0.415	2.83 ± 0.315	10.8 ± 1.40	3.09 ± 0.671	1.64 ± 0.334	1.82 ± 0.291	15.8 ± 3.10	4.03 ± 0.646	4.02 ± 0.544	4.74 ± 0.835
*CL_int,u,UGT_* (µL/min/mg)	222	277	334	344	40.3	326	421	313	324	952	1563	1288	47.3	164	177	164
*CL_int,u,UGT_* (mL/min/g of tissue)	NA	NA	NA	NA	1.61	13.0	16.9	12.5	4.15	12.18	20.0	16.5	0.974	3.38	3.64	3.39

Abbreviations: BSA, bovine serum albumin; CL_int,u,UGT_, unbound intrinsic clearance by glucuronidation; f_u,inc_, fraction unbound from protein in the incubation; HIM, human intestinal microsomes; HKM: human kidney microsomes; HLM, human liver microsomes; K_m_, Michaelis‐Menten constant; K_m,u_, unbound Michaelis‐Menten constant; NA, Not applicable; UGT, uridine diphosphate glucuronosyltransferase; V_max_, maximum velocity.

^a^ Mean ± standard error (n = 3).

^b^ Mean ± standard deviation (n = 3).

### 4.5 CYP inhibition potential

**Figure 5:**
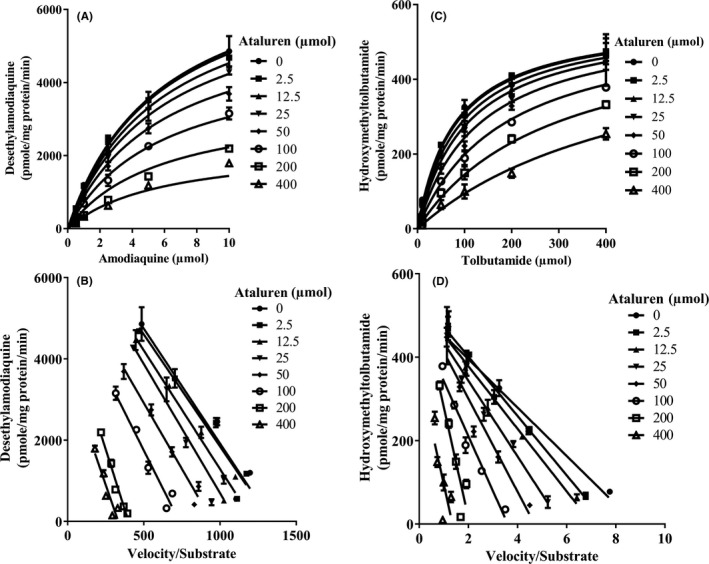
Inhibition of CYP2C8 (amodiaquine *N*‐deethylase, A & B) and CYP2C9 (tolbutamide methyl hydroxylase, C & D) activities by ataluren: noncompetitive (A), competitive (C) and Eadie‐Hofstee (B & D) plots (n = 3 for each data point)

The appropriate positive controls used in this study inhibited the enzyme activities at acceptable levels indicating that the test system was functional (data not shown). As summarized in Table S3, at concentrations up to 400 μmol/L (n = 3), ataluren did not inhibit enzyme activity for CYPs 1A2, 2B6, 2C19, 2D6, and 3A4/5. The IC_50_ values were estimated to be greater than 400 μmol/L, and due to solubility limitations, K_i_ values were not determined for these enzymes. Ataluren inhibited CYP2C8 and CYP2C9 activities with IC_50_ values of 165 µmol/L and 134 µmol/L, respectively. The mechanism study indicated that ataluren is a noncompetitive inhibitor of CYP2C8 with *K*_i_ (mean ± SE, n = 3) of 169 ± 6.19 µmol/L and is a competitive inhibitor of CYP2C9 with *K*_i_ (mean ± SE, n = 3) of 75.4 ± 4.42 µmol/L (Figure 5).

### 4.6 CYP induction potential

Hepatocytes from all three human donors used in the study responded well to exposure of all prototypical inducers. At the concentrations tested, ataluren was not cytotoxic as indicated by methylthiazolyldiphenyl‐tetrazolium bromide (MTT) assay (data not shown). Ataluren did not induce the activities of CYPs 1A2, 2A6, 2C8, 2C19, 2E1, or 3A4 relative to the vehicle control, 1% DMSO, but showed approximately 1.7‐ and 1.5‐fold induction (n = 3) over vehicle control at 400 µmol/L for CYP2B6 and CYP2C9 activity, respectively. The mean enzyme activities are summarized in Table S4.

## 5 DISCUSSION AND CONCLUSION

The findings of the current studies show that the direct conjugation of ataluren with glucuronic acid to form ataluren acyl glucuronide is the primary metabolic pathway of ataluren, consistent with in vivo observations.^20^ CYP plays only a minimal role in the metabolism of ataluren. UGT1A9 was the major enzyme responsible for the formation of ataluren‐*O‐*1β‐acyl glucuronide. UGT1A7 was found to be the minor enzyme for producing ataluren acyl glucuronide. Furthermore, in UDPGA fortified HLM, the loss of ataluren and the formation of ataluren acyl glucuronide correlated significantly with glucuronidation of propofol, a marker substrate for UGT1A9 activity. Human kidney microsomes were more active than liver microsomes and intestinal microsomes, whereas intestinal microsomes was least active in terms of unbound intrinsic clearance of ataluren glucuronidation.

Ataluren UGT reaction phenotyping results are similar to propofol glucuronidation in microsomes from human liver, intestine, and kidney.^26^ They are also consistent with the fact that UGT1A9 is mainly expressed in liver and kidney and is more abundant in kidney than in liver, is expressed at low levels in the small intestine and is not expressed in the lung.^28^ ^,^ ^29^ UGT1A7 is minimally expressed in the liver and at low levels in the kidney, small intestine, and similar to UGT1A9, it is not expressed in the lung.^28^ ^,^ ^29^

In vitro kinetic data revealed that human kidney microsomes is about 3.7‐fold more active than human liver microsomes in ataluren glucuronidation, in terms of CL_int,u_/mg protein, indicating that in addition to the liver, the kidney is also a key organ for the metabolism and disposition of ataluren in human. Though HIM showed relatively low CL_int,u_ for ataluren glucuronidation activity, contribution of intestinal UGTs cannot be excluded for ataluren first pass metabolism in small intestine following oral dose.

The addition of BSA enhances both CYP and UGT activities by sequestering fatty acids that are released from membranes.^30^ ^,^ ^31^ ^,^ ^32^ ^,^ ^33^ ^,^ ^34^ Overall, BSA has been shown to increase CL_int,u_, however, this effect is enzyme, tissue, substrate, and BSA concentration dependent. For example, BSA reduced K_m,u_ values, whereas having only a minor effect on V_max_ values for CYP2C9 ^30^ and UGT2B7.^31^ In contrast, BSA increased V_max_ in addition to lowering the K_m_ for UGT1A9 ^31^ and showed a minimal effect on UGT1A1 and UGT1A6.^32^ For two (buprenorphine and carvedilol) of the 10 compounds in HLM, 2% BSA reduced CL_int,u_.^33^ However, the results of the effect of BSA have been inconsistent. One study found that 2% BSA decreased apparent V_max_ for propofol‐*O*‐glucuronidation (UGT1A9), whereas increased V_max_ for zidovudine glucuronidation (UGT2B7) in HLM and in recombinant human UGTs.^34^

In this study, different concentrations of BSA (0%, 0.5%, 1%, and 2%) were used to evaluate the effect of BSA on ataluren glucuronidation using recombinant human UGT1A9 and human liver, kidney, and intestinal microsomes. Similar to results using propofol and 4‐methylumbelliferone as substrates,^32^ BSA increased total apparent K_m_ and protein binding in a BSA concentration‐dependent manner, and had minimal effect on V_max_. BSA increased CL_int,u_ due to the decrease in K_m,u_ in all tested systems at all BSA concentration tested. However, the maximal effect on CL_int,u_ were system/tissue dependent and were increased approximately 1.6‐, 10‐, 4.8‐, and 3.7‐fold for recombinant UGT1A9, and human liver, kidney, and intestine microsomes, respectively. Regarding the use of BSA, 2% BSA is most frequently used in different test systems. However, based on limited data, the BSA concentration may also play a role in enzyme activation in a substrate/system‐dependent manner.^32^ ^,^ ^35^ ^,^ ^36^ For ataluren, a benzoic acid derivative, 1% BSA was the optimal concentration, which is in agreement with Gill et al that 1% BSA was optimal for acids and 2% was the best for base/neutral.^26^ These results indicate that BSA titrations may be necessary for compounds with low depletion and high protein binding.

Ataluren did not inhibit enzyme activity for CYPs 1A2, 2B6, 2C19, 2D6, and 3A4/5 up to 400 μmol/L. Ataluren is a noncompetitive inhibitor of CYP2C8 with inhibition constant (*K*_i_) of 169 µmol/L and a competitive inhibitor of CYP2C9 with inhibition constant (*K*_i_) of 75.4 µmol/L. Free fractions of ataluren between 1 and 200 µmol/L were all close to unity in human liver microsomes at 0.1 mg/mL protein concentration (internal data). Therefore, the unbound inhibition constants (*K*_i,u_) are approximately equal to the respective total inhibition constants for both CYP2C8 and CYP2C9 under current incubation conditions. Following oral therapeutic doses of 10, 10, 20 mg/kg/day (morning, mid‐day and evening dose, respectively) in DMD patients, the mean C_max_ of ataluren at the steady state is around 79.9 μmol/L. Since the protein binding of ataluren in human plasma is high (99.6%), the free C_max_ of ataluren at the steady state is around 0.32 µmol/L (using free fraction value of 0.4% as measured) or 0.80 µmol/L (to be conservative assuming free fraction value of 1%), and thus is much lower than *K*_i,u_ values, 169 µmol/L for CYP2C8 and 75.4 µmol/L for CYP2C9. These data indicate that ataluren will likely not exhibit any clinically relevant effect due to CYP inhibition.

At concentrations up to 400 µmol/L in cultured primary human hepatocytes, ataluren did not induce the activities of CYPs 1A2, 2A6, 2C8, 2C19, 2E1 and 3A4, except for a mild 1.7‐ and 1.5‐fold induction over vehicle control at 400 µmol/L for CYP2B6 and CYP2C9 activity, respectively. This induction is considered not significant or clinically relevant, and thus, ataluren interaction with other concomitantly administered drugs due to enzyme induction is less likely.

In summary, results of in vitro metabolism studies showed that CYP‐mediated metabolism of ataluren is minimal, whereas acyl glucuronidation is the major metabolic pathway. UGT1A9 is the major enzyme that catalyzes glucuronidation of ataluren in liver and kidney. Enzyme kinetic studies indicated that in addition to the liver, the kidney is also a key organ for the metabolism and disposition of ataluren in humans, whereas other UGTs may be also involved in this reaction in the intestine. Ataluren showed no or little potential to inhibit or induce most of the CYP enzymes.

## DISCLOSURE

The study was sponsored by PTC Therapeutics. Authors are current employees of PTC Therapeutics and may hold stock or other equity positions with the company.

## AUTHOR'S CONTRIBUTIONS

RK, JM, EMW, and JB participated in research design; RK and JM conducted experiments; SH and YM synthesized ataluren‐*O*‐1β‐acyl glucuronide; RK, JM, EMW, JB, and NA performed data analysis, and RK, JM, EMW, JB, MW, JMC, NA, and EG wrote or contributed to the writing of the manuscript.

## ETHICS STATEMENT

All studies were conducted in accordance with all applicable ethical requirements.

## DATA REPOSITORY

Supplementary tables are listed in Appendix.

## Supporting information

Supplementary Material
