# Supplementary material for: In vitro metabolism, reaction phenotyping, enzyme kinetics, CYP inhibition and induction potential of ataluren
Source: Pharmacol Res Perspect. 2020 Mar 20;8(2):e00576. doi: 10.1002/prp2.576 (PMC7083565; doi:10.1002/prp2.576)
Supplement: Supplementary file 1 — Supplementary Material [file PRP2-8-e00576-s001.doc]

**Appendix**

**Supplementary** **Tables**

**Supplementary Table 1. Incubation conditions for ataluren CYP inhibition assays**

| CYP | Ataluren^†^ | | | |
| --- | --- | --- | --- | --- |
|  | Enzyme activity | Substrate conc.  (μM) | Protein conc. (mg/mL) | Incubation time (min) |
| 1A2 | Phenacetin *O*-deethylase | 70 | 0.1 | 7 |
| 2B6 | Bupropion hydroxylase | 25 | 0.1 | 7 |
| 2C8 | Amodiaquine *N*-deethylase | 1 | 0.1 | 7 |
| 2C9 | Tolbutamide hydroxylase | 50 | 0.1 | 7 |
| 2C19 | *S*-Mephenytoin 4′-hydroxylase | 40 | 0.1 | 7 |
| 2D6 | Dextromethorphan *O*-demethylase | 2 | 0.1 | 7 |
| 3A4/3A5 | Midazolam 1′-hydroxylase | 2 | 0.1 | 7 |
| 3A4/3A5 | Testosterone 6β-hydroxylase | 60 | 0.1 | 7 |

^†^ Ataluren was dissolved in DMSO with final concentrations at 0, 2.5, 12.5, 25, 50,100, 200 and 400 μM with 0.1% DMSO in final incubation.

CYP: cytochrome P450; DMSO: dimethyl sulfoxide;

**Supplementary Table 2. Analytical conditions for CYP inhibition and induction assays**

| CYP | Analyte | Ionization mode^†^*,*^‡^ | Analyte ion transition (*m*/*z*) |
| --- | --- | --- | --- |
| 1A2 | Acetaminophen | + ESI | 152→110 |
| 2B6 | Hydroxybupropion | + ESI | 256→167 |
| 2C8 | *N*-desethyl amodiaquine | + ESI | 328→283 |
| 2C9 | Methylhydroxy tolbutamide | + ESI | 287→171 |
| 2C19 | 4-Hydroxy mephenytoin | + ESI | 235→150 |
| 2D6 | Dextrophan | + ESI | 258→157 |
| 3A4/3A5 | 6β-Hydroxy testosterone | + ESI | 342→324 |
| 3A4/3A5 | 1’-Hydroxy midazolam | + ESI | 346→269 |
| 2A6 | 7-Hydroxycoumarin | - ESI | 161→133 |
| 2E1 | 6-Hydroxy chlorzoxazone | - ESI | 184→120 |

^†^ + ESI: electrospray in positive ionization mode;

^‡^ - ESI: electrospray in negative ionization mode.

**Supplementary Table 3. Inhibition of CYP enzyme activity by ataluren in pooled human liver microsomes**

| Activity assay | CYP | IC_50_ (µM, n=3) | K_i_ (µM, n=3) |
| --- | --- | --- | --- |
| Phenacetin *O*-deethylase | CYP1A2 | >400 | ND |
| Bupropion hydroxylase | CYP2B6 | >400 | ND |
| Amodiaquine *N*-deethylase | CYP2C8 | 163  (150 to 177) ^‡^ | 169±6.19^†^ (157 to 181) ^‡^ |
| Diclofenac 4′-hydroxylase | CYP2C9 | 135  (129 to 141) ^‡^ | 75.4±4.42^†^ (66.7 to 84.2) ^‡^ |
| *S*-Mephenytoin 4′-hydroxylase | CYP2C19 | >400 | ND |
| Dextromethorphan *O*-demethylase | CYP2D6 | >400 | ND |
| Testosterone 6β-hydroxylase | CYP3A4/5 | >400 | ND |
| Midazolam 1′-hydroxylase | CYP3A4/5 | >400 | ND |

^†^Mean ± standard error (n=3); ^‡^ 95% confidence interval; CYP: cytochrome P450; IC_50_: concentration of an inhibitor that causes a 50% decrease in enzyme activity for an enzyme; K_i_: inhibition constant; ND: not determined.

**Supplementary Table 4. Induction of CYP enzyme activity by ataluren in primary human hepatocytes (n=3)**

| Activity assay | CYP | Ataluren (µM) | Vehicle control % (n=3) | | Positive control % (n=3) | |
| --- | --- | --- | --- | --- | --- | --- |
|  |  |  | Mean | SD | Mean | SD |
| Phenacetin *O*-deethylase | CYP1A2 | 4 | 118 | 11.0 | 1.32 | 0.788 |
|  |  | 40 | 116 | 11.5 | 1.24 | 0.967 |
|  |  | 400 | 100 | 26.1 | 0.087 | 1.89 |
| Coumarin 7-hydroxylase | CYP2A6 | 4 | 100 | 12.6 | NC | NA |
|  |  | 40 | 111 | 3.79 | NC | NA |
|  |  | 400 | 118 | 17.9 | NC | NA |
| Bupropion-hydroxylase | CYP2B6 | 4 | 111 | 17.4 | 2.81 | 4.27 |
|  |  | 40 | 123 | 8.96 | 6.27 | 2.07 |
|  |  | 400 | 170 | 16.5 | 19.8 | 6.80 |
| Amodiaquine *N*-deethylase | CYP2C8 | 4 | 93.0 | 13.4 | NC | NA |
|  |  | 40 | 109 | 7.00 | NC | NA |
|  |  | 400 | 67.6 | 11.3 | NC | NA |
| Tobultamide methylhydroxylase | CYP2C9 | 4 | 100 | 11.8 | NC | NA |
|  |  | 40 | 109 | 10.3 | NC | NA |
|  |  | 400 | 146 | 29.7 | NC | NA |
| *S*-Mephenytoin 4′-hydroxylase | CYP2C19 | 4 | 112 | 31.4 | NC | NA |
|  |  | 40 | 103 | 17.0 | NC | NA |
|  |  | 400 | 99.0 | 12.0 | NC | NA |
| Chlorzoxazone 6-hydroxylase | CYP2E1 | 4 | 99.0 | 5.34 | NC | NA |
|  |  | 40 | 109 | 12.4 | NC | NA |
|  |  | 400 | 105 | 17.7 | NC | NA |
| Testosterone 6β-hydroxylase | CYP3A4/5 | 4 | 103 | 2.52 | 0.594 | 0.547 |
|  |  | 40 | 105 | 4.04 | 1.52 | 2.08 |
|  |  | 400 | 109 | 14.5 | 3.72 | 5.03 |

CYP: cytochrome P450; NA: not applicable; NC: not calculated; SD: standard deviation.
